# Try to see it my way: exploring the co-design of visual presentations of wellbeing through a workshop process

**DOI:** 10.1177/1757913919835231

**Published:** 2019-04-08

**Authors:** MP Craven, R Goodwin, M Rawsthorne, D Butler, P Waddingham, S Brown, M Jamieson

**Affiliations:** NIHR MindTech MedTech Co-operative, Institute of Mental Health, University of Nottingham Innovation Park, Jubilee Campus, Triumph Road, Nottingham NG7 2TU, UK; Bioengineering Research Group, Faculty of Engineering, University of Nottingham, Nottingham, UK; The Oliver Zangwill Centre, Cambridgeshire Community Services NHS Trust, Ely, UK; NIHR Collaboration for Leadership in Applied Health Research and Care (CLAHRC) East Midlands, Institute of Mental Health, Nottingham, UK; NIHR MindTech MedTech Co-operative, Institute of Mental Health, University of Nottingham Innovation Park, Jubilee Campus, Nottingham, UK; The Oliver Zangwill Centre, Cambridgeshire Community Services NHS Trust, Ely, UK; NIHR MindTech MedTech Co-operative, Institute of Mental Health, University of Nottingham Innovation Park, Jubilee Campus, Nottingham, UK; Division of Psychiatry and Applied Psychology, University of Nottingham, Jubilee Campus, Nottingham, UK; Institute of Health and Wellbeing, Administration Building, Gartnavel Royal Hospital, Glasgow, UK

**Keywords:** mental health, digital health, self-management, visualisation, public health

## Abstract

**Aims:**

A 10-month project funded by the NewMind network sought to develop the specification of a visualisation toolbox that could be applied on digital platforms (web- or app-based) to support adults with lived experience of mental health difficulties to present and track their personal wellbeing in a multi-media format.

**Methods:**

A participant co-design methodology, Double Diamond from the Design Council (Great Britain), was used consisting of four phases: Discover – a set of literature and app searches of wellbeing and health visualisation material; Define – an initial workshop with participants with lived experience of mental health problems to discuss wellbeing and visualisation techniques and to share personal visualisations; Develop – a second workshop to add detail to personal visualisations, for example, forms of media to be employed, degree of control over sharing; and Deliver – to disseminate the learning from the exercise.

**Results:**

Two design workshops were held in December 2017 and April 2018 with 13 and 12 experts-by-experience involved, respectively, including two peer researchers (co-authors) and two individual-carer dyads in each workshop, with over 50% of those being present in both workshops. A total of 20 detailed visualisations were produced, the majority focusing on highly personal and detailed presentations of wellbeing.

**Discussion:**

While participants concurred on a range of typical dimensions of wellbeing, the individual visualisations generated were in contrast to the techniques currently employed by existing digital wellbeing apps and there was a great diversity in preference for different visualisation types. Participants considered personal visualisations to be useful as self-administered interventions or as a step towards seeking help, as well as being tools for self-appraisal.

**Conclusion:**

The results suggest that an authoring approach using existing apps may provide the high degree of flexibility required. Training on such tools, delivered via a module on a recovery college course, could be offered.

## Introduction

Digital technology is readily available in the hands of many individuals. It holds promise for supporting improved public health through the exploitation of its sophisticated and user-friendly multimedia functions as well as its always-on connectivity. The deployment of digital technology in healthcare, typically termed e-Health (electronic) and more recently m-Health (mobile), is to be found on platforms such as personal computers, smartphones, tablet and wearables.

In recent years, a strong focus on digital mental health has emerged.^1^ In the UK, the NewMind network,^2^ funded by the Engineering and Physical Sciences Research Council (EPSRC), was founded to explore ‘the potential for technology to transform the management & treatment of mental health conditions, whilst seeking to address underlying EPS research challenges’. The definition of mental health in the national ‘No Health without Mental Health’ policy is that it is a positive state of mind and body, feeling safe and able to cope, with a sense of connection with people, communities and the wider environment.^3^ Levels of mental health are influenced by the conditions people are born into, grow up in, live and work in.

A NewMind roadmap was published identifying relevant health-related goals, for example, supporting community and patient self-management, and more personalised services and the important technology areas aligned to those, for example, design of human-centric systems. Events were held to encourage the initiation of collaborative projects. This article results from one such project, conceived in February 2017, focusing on visualisation of wellbeing.

The NewMind Dynamic Visualisation project aimed to explore the area of graphical, and more broadly multi-sensorial, technologies that could be applied to an individual living with lived experience to record, communicate, track or otherwise help manage their wellbeing through digital media representations.

Wellbeing is understood and measured in many different ways. The five-item World Health Organization Well-Being Index (WHO-5)^4^ is among the most widely used questionnaires assessing subjective overall wellbeing, covering aspects of physical and mental health. The WHO-5 is based on the theory that the following steps will help to achieve wellbeing: connect, be active, take notice, keep learning and give. Numerous wellbeing measures focus on psychological wellbeing, such as the Warwick-Edinburgh Mental Well-Being Scale (WEMWBS)^5^ which aims to capture a wide conception of wellbeing, including affective-emotional aspects, cognitive-evaluative dimensions and psychological functioning. Another frequently used wellbeing measure, Diener’s Satisfaction with Life Scale (SWLS),^6^ measures global cognitive judgements of satisfaction with one’s life. Furthermore, there are many more wellbeing definitions and theories, so part of this project, as described below, was to perform a literature review, accumulate constructs of wellbeing and see which resonated best with our workshop participants.

The subject was suggested by Cambridgeshire Community Services National Health Service (NHS) Trust practitioners who primarily work with persons recovering from brain injury and was developed in conjunction with researchers and experts-by-experience at the Institute of Mental Health (a research centre jointly run by the University of Nottingham and Nottinghamshire Healthcare NHS Foundation Trust, which focuses on psychosocial interventions) and with additional human computer interaction (HCI) expertise from the University of Glasgow. The idea was selected for funding after short-listing and a ‘dragon’s den’ style competition, which included scientific and peer panellists. A total of £15,000 funding was made available to run a 10-month project from August 2017 to May 2018.

Relevant background to the project was the knowledge that outcome measures are routinely completed as part of a service user (SU) assessment within a mental health setting, but these are either not routinely relayed^7^ or when provided, it is unclear how meaningful they are to the individual.^8^ Responding to this information asymmetry, giving users information in a way that is relevant to them should contribute to empowerment.^9^ Greater personalisation may help address the low adherence common to digital health products.^10^ In addition, tacit knowledge was available within the team about smartphone apps which use a variety of visual techniques to present and track health and wellbeing, but this use had not been analysed. In the initial sandpit, experts-by-experience suggested individualising their wellbeing requirements would allow them to better manage their health condition, for example, help them identify triggers and patterns impacting on their individualised wellbeing or impacting on other symptoms. A longer-term goal for the project was judged to be provision of design advice for app developers.

## Methods

From the start, the project team was committed to a person-based approach.^11^ Indeed, our team included two peer researchers from the beginning and we wished to choose a methodology that would make the most of the creativity of a larger number of experts-by-experience. The Double Diamond co-design process from the British Design Council^12^ was ideal for this project, using a two-stage engagement with end-users, enabling creativity through divergence followed by convergence on consensus in each stage. The first diamond consists of two phases; Discover and Define, and the second diamond consists of two further phases; Develop and Deliver. For this project, the four phases mapped well on to discovery through rapid reviews of the literature and of existing technologies by the research team, followed by definition with experts-by-experience in an initial workshop format. The second diamond enabled ideas to be refined in a follow-up workshop and the end-point was to be a specification or design guidelines.

### Discover – literature and app searches

The Discover phase consisted of three linked searches to find: (1) evidence-based material on wellbeing, (2) visualisation tools developed for clinical use and emerging ideas from the HCI domain and (3) visualisation techniques being offered in current digital products. The wellbeing literature was subjected to a structured literature review, the tools and technology literature was conducted as an opportunistic search, since it involved conference papers and websites as well as academic literature, and a product review was conducted by searching the Google Play Store (top 100 health apps) and the beta version of the new UK NHS^13^ app library from which 10 apps in the mental health category were identified (during November 2017). The review methodology proceeded by separate chunking of the wellbeing literature and of the combined visualisation and app search findings and these were transferred on to two sets of slides for the first workshop.

### Define – co-design workshop 1

Workshop 1 first involved a free discussion of wellbeing concepts facilitated by Clean Language questioning to avoid forcing interpretations from the literature at the start^14^ before proceeding to presentation of findings from the searches. The feedback from participants was used to enhance the thematic model of key wellbeing concepts. The rest of the workshop was divided into two sections, one to consider wellbeing terms presented as words and one to consider visualisation terms presented as words with explanatory graphics. Both sections were preceded by an overview of the terms from the literature, by means of a slideshow, and an explanation of the rating task to be conducted. All of the invited participants were asked to complete a personal workbook in which were listed all of the wellbeing and visualisation terms from the literature, with each term displayed on a single page of the workbook to allow it to be considered in turn. Opinions from the group were captured through individuals marking items of personal relevance in their own workbook (Yes, Maybe or No) and they were also asked to highlight one or two of the most personally important wellbeing categories and preferred visualisation techniques by marking them with single asterisk (referred to later in this article as a ‘Star’ rating). Participants were also encouraged to suggest new wellbeing and/or visualisation themes if they perceived any relevant to them were missing. Additional field notes were taken by the facilitators and the interim results were analysed and fed into the second workshop.

### Define – co-design workshop 2

Workshop 2 involved presenting the summary of results from Workshop 1, followed by a set of animated images (GIFs) found on the web to help illustrate the visualisation ideas already generated to inspire broader thinking on digital presentation possibilities. Specific guidance on developing visualisations in more detail was given alongside a talk by one of the peer researchers as an example of how they would approach developing their own visualisation based on the guidance. Participants were given flipchart paper, sticky notes of various colours, coloured pens and scissors to develop and draw their personal visual representations over 1 h. Facilitators from the project team were on hand to give further prompts about the key design considerations (listed later) to help them draw pictures if necessary, and to take notes and photograph results.

Although formal approval was not deemed necessary (see below), participant information sheets and a consent form (including for use of photographic material) were used for each workshop. In addition, ground rules were discussed at the start of each workshop to agree an open and non-judgmental environment for the discussion. Preparation for each workshop involved the research team doing the exercises themselves beforehand to ensure comprehensibility of content, ease of engagement with the material and test timings. It also included ensuring access for one participant with visual impairment such that the exercises were given text descriptions and tested on screen reader software.

### Deliver – dissemination activities

The Delivery phase consisted (to date) of dissemination activities including a presentation at the Institute of Mental Health annual research day with some of the workshop participants being present and by sending the results to participants.

## Results

The pair of workshops were held on December 2017 and April 2018 with 13 and 12 experts-by-experience, respectively, including the two peer researchers in our project team. A total of seven participants were present in both workshops, providing a degree of continuity. In both workshops, there were two carer-service user dyads with one dyad participating in both workshops.

In Workshop 1, the relevance of wellbeing constructs and existing visualisation techniques were explored with experts-by-experience after preparation by the project team from the searches and some initial analysis to ‘chunk’ these into manageable sets for deliberation over. In Workshop 2, 20 detailed visualisations were produced, the majority focussing on detailed presentations of individual wellbeing. Overall, users’ engagement was positive. One quote from a message after Workshop 1 was, ‘just wanted to say thank you for the meeting today. I learnt a lot and met some great people. Best of all it made me feel like me again and I can’t thank you enough for that’.

### Discover and define phases

#### Searches

As mentioned previously in the Methods section, the wellbeing terms presented in Workshop 1 were derived from a structured literature search (details and references omitted for reason of space) that resulted in 45 papers from which 89 wellbeing terms contained within them were thematically clustered to give 17 top-level themes having two to five subsections in each are as follows:

1. Close relationships: marriage, social support, childhood family, family unit and friends.
2. Part of community: be respected, belonging, contribute to others and safety.
3. Wider connection: spirituality and religion.
4. Good mood: happiness, being calm, being well-rested and having positive feelings.
5. Life satisfaction: gratitude, enjoyment, confidence and vitality.
6. Negative feelings: stress, anxiety, adverse life events, pain and anger.
7. Depressed mood: loneliness, distress, guilt and worthlessness.
8. Perseverance: flexibility, resilience, hope, humour and optimism.
9. Relationship to self: self-acceptance, identity, self-esteem and self-forgiveness.
10. Control: agency, autonomy, purpose and meaning of life.
11. Ability: achievement, competence, learning and growth.
12. Thinking skills: problem solving, decision making, concentration and memory.
13. Job: income, security, satisfaction and volunteering.
14. Social context: age, education, living conditions, home ownership and governance.
15. Interest: creativity, culture, sport and natural environment.
16. Physical health: conditions, mobility, sleep, fatigue and diet.
17. Functional activity: household roles, self-care, healthy lifestyle, leisure and transport.

**Figure 1:**
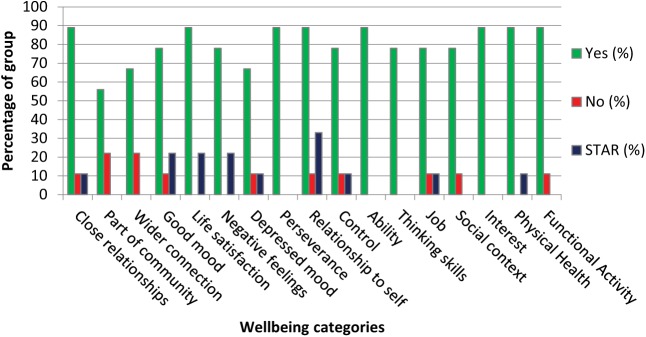
Endorsement (Yes or No, STAR = highly endorsed) of wellbeing terms within the Workshop 1 group (*N* = 11)

The results from the Workshop from the initial exercise of scoring the 17 themes are shown in Figure 1, presenting the percentages of Yes and No ratings (Maybe and blank responses omitted for clarity) and the percentage of themes given ‘Star’ ratings, that is, one or two themes marked by an asterisk as being most relevant. There was a high degree of personal recognition of relevance for all of the terms, and there was a spread of highly endorsed themes (9 of the 17 themes were highly endorsed by up to 3 of the 11 respondents). Field notes from the free discussion were also analysed for emergent themes. Additions included growth (kindness, curiosity, compassion, mindfulness); communication (understand others, be understood); relationship to other people (suspicion, paranoia); activism (resisting injustice, fighting for self); and compassion (healing self, healing others).

The visualisation literature search was opportunistic across all health conditions and included quality of life, dimensions of physical health (e.g. pain, fatigue) as well as psychological or emotional states. The app search inclusion criteria were that mental health or wellbeing data was presented to the user and that the app was available to download without login access. The literature and app store searches products, taken together and clustered into a draft taxonomy, yielded nine main visualisation types from 45 papers and 27 apps. The first three types were based on the common distinction between scales, ordinal and nominal data types (as used in SPSS statistical software, for example), and a further six representation types were identified:

1. Numerical/interval: graphs, colour spectrum, rating scales, abstract shapes varying in amount of shading or size.
2. Ordinal (including binary): ordered iconic or pictorial progressions not on an interval scale.
3. Nominal/symbolic: iconic or pictorial without ordering.
4. Multidimensional: stars, RADAR diagrams.
5. Geographical: maps, distance measures.
6. Gestural and/or postural.
7. Textural, tactile or thermal.
8. Metaphorical.
9. Musical.

Numerical or interval representations included visual analogue scales,^15^ various line, dot or bar graphs, colour spectrum^16^ and clickable buttons with audio.^17^ Ordinal type included scales with pictures or icons such as faces^18–20^ focusing on various physical aspects of wellbeing. Stark QoL^21^ used a set of two- and three-point picture scales to visualise mood, degree of social contact and energy (fatigue). The Cantril Self-anchoring Striving Scale used position on a ladder.^22^ Scales with more abstract shapes included circles of varying size, fill or shading of circles^23,24^ or wavy lines with varying amplitude, period, sharpness and speed of movement.^25^ Nominal/symbolic type included sets of emojis^26^ (such as ☺) or emoticon text symbols. Multidimensional type included RADAR or star-shaped diagrams.^27,28^ Geographical type used distance, separation, trajectory or position of objects on a map.^29,30^

Gestural/postural type was found in virtual reality software that used animated manikins in a compassion therapy study.^31^ Textural, tactile or thermal types included a smartphone contact list enhanced with textural graphics, vibrations and sound to represent a person’s mood^32^ and one example of heat stimulus.^33^ Metaphorical type^34^ examples were a personal rain-cloud, weighted down bedclothes and locked doors. Various animated metaphors were seen, for example, the mental health awareness game Elude uses imagery of climbing trees, floating on leaves or clouds and of being trapped underground.^35^ A metaphor based on energy fields was found (as an aspect of Rogerian psychotherapy). Musical type was seen in a few studies that attempted to map mood or feelings to music selection choices^36^ or to explore the effect of music on mood.^37^

Many (23 of 27) examples of existing mental health apps in the online search results used visualisation techniques during input (21 apps), display (19 apps) or both (14 apps). Mood-tracking was most commonly represented with an emoji with eight apps in total, seven combining this with colour. Several apps that tracked or assessed anxiety and depression afforded input as a slider to rate symptom strength or agreement with questions asked (five apps). Apps that tracked mental health over time mostly displayed this graphically, with the *x*-axis representing time and the *y*-axis representing mood state or mental health score (seven apps) whereas apps that assessed mental health state in more detail across a number of dimensions tended to display a fuel gauge or a RADAR diagram. In addition, some apps used the information entered to offer advice or support, pairing user to a therapist, presenting information about mental health services, asking for follow-up or making suggestions about coping techniques. Some used gamification techniques to improve engagement with health tracking and to encourage behaviour change.

**Figure 2:**
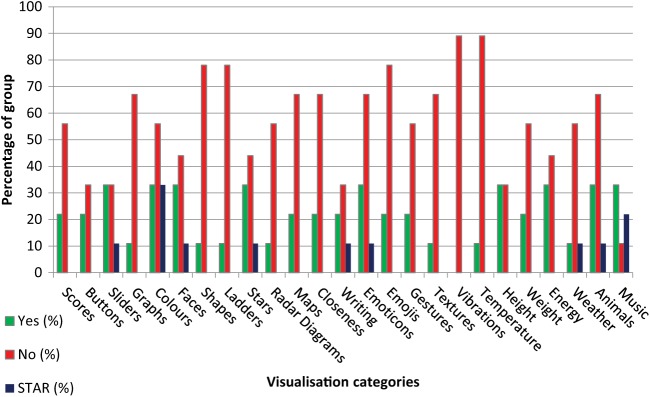
Endorsement (Yes or No, STAR = highly endorsed) of selected visualisation types and sub-types within the Workshop 1 group (*N* = 11)

As with the wellbeing literature, the visualisation search findings were used to produce material for Workshop 1 to discuss these with experts-by-experience including another exercise to examine preferences, where individuals were asked to make personal Yes/No/Maybe selections for liking each visualisation type and to highlight with a ‘Star’ one or two of the most liked, with results as shown in Figure 2 (‘Maybe’ ratings again omitted for clarity). It can be seen from examining Figure 2 that for all of the visualisation types except music, the preferences were at best an even split between ‘Yes’ and ‘No’ or were disliked by a majority of the group. Top group preferences were seen for sliders, colours, faces, stars, emoticons, weather, animals and music. It is noteworthy, considering that these are often found in currently available apps, that graph visualisations were disliked by over 65% of the group and nearly 80% disliked emojis (noting that emojis could be used for either selection or for display but we did not distinguish between these functions). Tactile (vibrations) and thermal ‘visualisations’ were the most disliked types.

#### Group work

After the presentation of the search results by the project team, participants were encouraged to discuss visualisations in small groups and to offer personal examples. Some aligned to the draft taxonomy. Ordinal type included binaries of love-hate, do-don’t and gender, plus an inverted triangle/pyramid of mood. Rankings of activity and self-care were offered. Nominal/symbolic type included colour saturation, a spiritual symbol ‘Om’ and personalised cartoons. A multidimensional layered heart model was drawn. An added theme was Thought Processing: speed of thinking, perception of time, noisiness (number of voices). A large number of specific metaphors were described as follows: weather; nature (‘sea of thoughts’, trees); navigation (‘sat-nav’); ‘happy’ spaces (cathedral, holidays and jousting event); animals (dog, horses); and protection and safety (locking/shutting, armour).

### Develop phase

**Figure 3:**
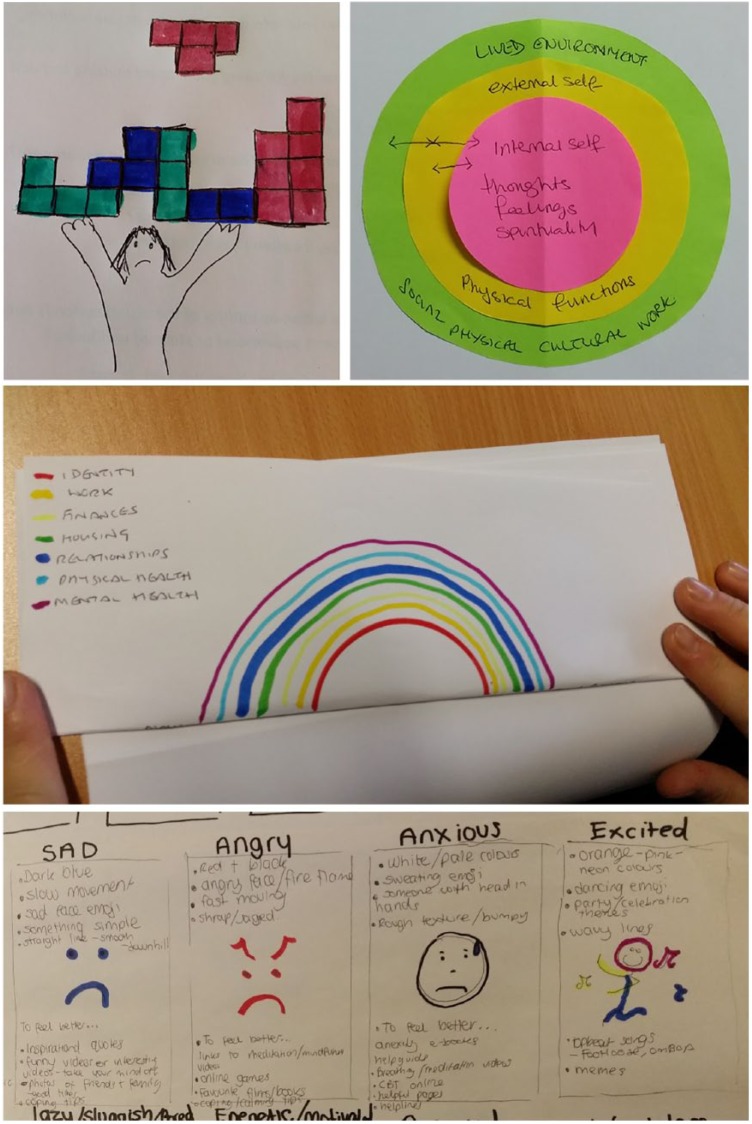
Example visualisations from Workshop 2

In Workshop 2, participants developed ideas in more detail including the peer researchers in our project team. Others of the project team took notes and took photographs as before. Figure 3 shows a few extracts from the set of 20 visualisations produced.

Table 1 shows selected results from the workshop, highlighting the media used or proposed and identifying the visualisation types and variations used to show differences in wellbeing. In addition to the tabulated results, participants expressed various ideas for managing the prompting of visualisation to themselves and for sharing the visualisations with third parties, similar to other self-management tools such as Copeland’s Wellness Recovery Action Plan^38^ but adapted for digital context. These included opting in or out of prompting and sharing (including automated daily reporting), having the app ask daily about mood or prompt viewing of visualisations, sharing ratings with family, general practitioner (GP) or consultant, providing alerts for extremes of measures and reminders about ‘truths’ (even if undesirable), pointing the individual to targeted sources of advice at a time of difficulty or contacting a third party to provide assistance.

**Table 1:** 

Selected visualisations from participants in Workshop 2 (from *N* = 12)		
Description	Media	Main visualisation type(s)
‘Dancing graph’ with audio soundtrack to reflect mood.	Graph of the audio signal.	Numerical: intensity of the graph’s movement and sound.
Picture of self/wellbeing as a balance in three areas: internal self (thoughts, feelings, spirituality), external self (physical functions); lived environment (social, physical, cultural, work). Also pictured in Figure 3.	Series of concentric discs of different colours. Coloured with a range of saturation/intensities (fading to white). May add intensity of sound.	Ordinal: degree to which discs are occupied. Dimensions of agency and sense-making, and capacity to learn or grow. Colour: well state would have a moderate saturation. Hyper or hypo saturation at the extremes. Extremes indicate warning signals or help identify a justifiable situation.
Set of 15 emotion cards: happy, sad, angry, anxious, excited, optimistic, depressed, insecure, shame and so on. Also pictured in Figure 3.	Use of detailed imagery for each emotion. Meaningful use of colour, texture, expression and gesture.	Symbolic: head in hands, dancing, sweating, worry lines.
Personal app design.	Babies, music, comedy, work, challenge.	Multidimensional: laughing to crying. Loud & lively to sad & slow, and coping with attending to not attending. Thinking of a challenge to achieving challenge.
Map of life, death, hope and despair.	Imagery of a flower, sun and rain to represent hope, happiness and needs met versus despair and sadness, where needs are not met.	Metaphorical: alive as a flower growing in nurturing sun and rain, dead as a neglected drooped flower.
Putting a carer’s intrusive thoughts outside, while they stay inside without hostility. Thoughts are mindlessly picking grass outside.	Drawing or animation of door between inside and outside. Staged over a period time. Sound of door open and closing.	Geographical: degree of closure and distance from door. Also Ordinal: using variation of noise (near/gentle to far/noisy) and Numerical: amount of grass picked.
‘Haptic Pad’ wearable on wrist or waist which pokes, grabs, slaps, strokes or soothes according to mood.	Tactile feedback.	Tactile: feelings vary from knotting of hands to grabbing the stomach.

## Discussion

From Workshop 1, as seen in Figures 1 and 2, while participants concurred on the range of typical dimensions of wellbeing, there were marked differences in preference for the types of visualisations on offer in the literature and in existing apps. From the results of the group work session, the highly personal nature of the visualisations generated and their interpretation were in contrast to the techniques currently on offer. The example of a suit of armour was variously used by different participants to describe feeling well and strong (jousting), feeling safe (protection) and feeling tired (fatigue and restricted movement).

In Workshop 2, the visualisation types suggested are seen in the literature and in existing apps. However, the subject matter of the visualisations and their modes of variation, as seen in Table 1, were mostly bespoke and are not currently supported in existing apps.

In the process of discussion with end-users, it was seen that lines were often blurred between the usefulness of visualisation tools for either self-appraisal or as a self-managed intervention, or both. O’Hanlon and Bertolino^39^ stress the importance of personal ‘fit’ of any intervention such that self-appraisal can lead to recognition and become an intervention. The immediacy of deployment, imagined to be deliverable on a personal mobile device, may have lent itself to a more proactive self-appraisal. A few of the design ideas developed by participants included sharing with clinical professionals where a visualisation can be considered to be a first step in recognising need for help or advice.

The strength of this study is that it was conducted as set of patient and public involvement (PPI) events which invited meaningful co-production. The mixture of group and individual activity in a safe peer group setting may have contributed to the large amount of creative output freely offered.^40^ Group sizes were quite small, although not unusual for a workshop format, but the participants seemed representative of SUs and individuals in recovery or recovered. The workshop had been advertised to attract persons with an association with mental health difficulties and many of the individuals disclosed their condition as part of the discussion about wellbeing. We are, therefore, confident that the group was a naturalistic sample of persons of different ages (younger and older adults), gender and ethnicity who had experience of past or ongoing mental health difficulties in the community setting. Disclosed conditions included depression, anxiety, bipolar disorder, schizophrenia and borderline personality disorder. Personal experiences of mental health recovery were enhanced by insights from carers.

As seen in the results, almost all of the presented wellbeing constructs were of relevance to the majority, as seen in Figure 1, which is perhaps not surprising coming from the established literature. However, the visualisation preferences were much more subjective. We cannot exclude the possibility of confirmation bias from these Workshop 1 exercises, but the difference in overall response to the wellbeing constructs and the visualisation types remains. Furthermore, the results may have been different in other groups, but the strength of opinion evidenced in Figure 2 may well be replicated by another group, albeit with different preferences.

## Conclusion

Due to the nature of the visualisations produced by the groups of experts-by-experience, as we continue the Deliver phase of our NewMind project, the view of the project team has changed somewhat from its preconception about designing an app toolbox. This is because the visualisations created were so complex and highly personal and also due to the preferences for existing visualisation tools being so diverse. It would need a study with many more participants to provide designers with a toolbox of constructs that would be useful for ‘most’ people. Such an approach might still not be worthwhile because of the level of idiosyncrasy displayed even with a small number of participants as seen here. This conclusion is strengthened by the fact that the graphics tools app developers currently draw from (graphs, emojis etc.) may not necessarily be meeting the needs of potential users; our results suggest that many visualisation choices would have to be offered in a single app to please a majority.

We are now of the opinion that an authoring approach may be more appropriate, where support for user-generated content is provided by more general digital applications and individuals elect to use these to create or customise their own visualisations, rather than pick from a set of predefined choices. In recent years, authoring has moved from the desktop PC to mobile devices. Examples of applications that already exist are MentalSnapp (a video-diary approach) and BitMoji which allows the creation of personalised scenes that a user can place themselves in as an ‘avatar’ character. We note that embodied virtual agents have recently been suggested for personalising visual feedback.^41^ In addition, messaging apps such as WhatsApp and Snapchat allow easy communication of videos and animations as well as text, pictures and emojis and provide enhancement tools for generating these. Less private platforms include YouTube and Facebook for communicating personal videos and photographs. We plan to discuss these delivery choices further with experts-by-experience.

Some of the project team already have experience with delivering a training course on the use of digital tools (websites, social media and games) for recovery, and so, one public delivery mechanism could be as an add-on session to this kind of course. Outstanding challenges include how well individually chosen measures may correlate with clinical measures or the degree to which people self-managing health would be prepared to actively complete both. Successful implementation of dynamic visualisation could enable predictive analysis of sustained wellbeing and potential relapse, facilitating ‘lean consumption’^42^ of scarce health and care resources.
